# Significance of climate change in the emergence of human fascioliasis in Upper Egypt

**DOI:** 10.1186/s40794-024-00234-z

**Published:** 2024-12-01

**Authors:** Naglaa Zanaty, Nagat Ibrahim, Haidi Karam-Allah Ramadan, Alzahraa Abdelraouf Ahmad, Amal Saad-Hussein

**Affiliations:** 1 Department of Environmental Studies, National Authority for Remote Sensing and Space Sciences, Cairo, Egypt; 2 Department of Gastroenterology, Hepatology, and Infectious Diseases, Assiut Liver Center, Ministry of Health, Assiut, Egypt; 3 Department of Tropical Medicine and Gastroenterology, Faculty of Medicine, Assiut University, Assiut, 71515 Egypt; 4 Department of Medical Parasitology, Faculty of Medicine, Assiut University, Assiut, 71515 Egypt; 5 Department of Environmental and Occupational Medicine, Environment and Climate Change Research Institute, National Research Centre, Cairo, Egypt

**Keywords:** *Fasciola*, Climate, Humidity, Temperature, Upper Egypt

## Abstract

**Background:**

Climate change in the upcoming years will raise the health burden of zoonotic parasites. As a liver fluke, *Fasciola* depends on certain climate conditions to complete its life cycle and is significantly influenced by climate changes. We aimed to investigate the relationship between the increasing prevalence of human fascioliasis and climate changes in Upper Egypt.

**Methods:**

Records of *Fasciola* cases in Assiut Governorate in Upper Egypt were evaluated between September 2018 and March 2023. The annual and monthly climate parameters of the region’s temperature and humidity acquired from ERA5 and FLDAS were investigated between 2000 and 2023.

**Results:**

A total of 303 patients were included. The mean age was 33.9 ± 17.4 years; 57.1% were females, and the majority were rural residents. Positive correlations were found between temperature and the recorded cases in 2018, 2020, 2021, and 2022 (*r* = 0.92, 0.41, 0.61, and 0.60, respectively). In 2018 and 2022, humidity and *Fasciola* frequency had a significant positive correlation (*r* = 0.97 and 0.49, respectively). An outbreak of fascioliasis was recorded in September 2018, coinciding with the peak temperature and high humidity levels, exceeding the average climatology range from 2000 to 2017. The recorded cases exhibited a seasonal pattern, with peaks in hot, humid summer and autumn.

**Conclusion:**

The rise of human fascioliasis in Upper Egypt is influenced by local climate characteristics. A climate-based map of *Fasciola* distribution using forecast risk models is needed to predict future outbreaks and for better control.

**Supplementary Information:**

The online version contains supplementary material available at 10.1186/s40794-024-00234-z.

## Introduction

*Fasciola hepatica* and *F. gigantica* cause a zoonotic disease affecting the liver and biliary ducts of humans and ruminants, leading to chronic hepatitis, obstructive jaundice, and cholangitis. This significantly impacts global livestock productivity, causing substantial economic losses. *Fasciola* is widely distributed geographically and represents a major parasitic disease [1, 2]. It undergoes different developmental stages and requires specific climatic and environmental conditions to complete its lifecycle. The infected hosts excrete eggs that hatch in water, releasing miracidia which infect snails. In snails, the parasites develop and release cercariae, which encyst on vegetation. Infection occurs through consuming plants containing these metacercariae [3].

The life cycle of the parasite *Fasciola hepatica* is greatly influenced by temperature and rainfall. In regions with an average temperature above 10°C for more than six months annually, the parasite becomes endemic, with a reported summer/winter cyclic pattern for snail infection [4]. However, in warmer climates, rainfall and moisture become limiting factors. Other factors such as soil properties [5], vegetation, and altitude may also influence the parasite’s life cycle [6]. Permanent water sources and irrigation activities restrict the transmission of *Fasciola* to the wet season only [7]. Based on the United Nations Intergovernmental Panel on Climate Change (IPCC) findings, a rise in global temperatures by 1.8°C to 4.0°C is predicted in the next 90 years [8]. Increasing temperatures and changes in moisture levels can affect the transmission dynamics and distribution of *Fasciola*. The parasite’s free-living stages and snail host require high moisture to complete their life cycle. So changes in moisture and humidity can influence the timing and frequency of *Fasciola* outbreaks [9].

Climate change and environmental factors have been widely reported to affect *Fasciola* developmental stages and snail vectors [10]. For instance, in the past years, there has been a progressive increase in rainfall in some parts of the Mediterranean region, resulting in a severe outbreak of acute fascioliasis in sheep in southern Italy [11] and in the UK [12]. Therefore, changes in environmental conditions can influence disease dynamics by supporting the growth of overwintering larvae [13]. Very few studies have investigated the impact of climate change on human fascioliasis. A recent review found that temperature variations significantly affected snail populations, parasite burden, and disease spread [14]. Hence, understanding this impact can help with prevention and control programs for fascioliasis, which has been reported as a re-emerging or emerging parasitic disease in various nations [15].

In the last 30 years, fascioliasis has become endemic in Egypt, with mild to severe cases reported, especially in the Nile Delta region. The estimated infection rate in Egypt is about 5.7%. Cases in Upper Egypt are less common and often go unreported due to a lack of screening studies [16] and the subclinical nature of most cases [17, 18]. In Egypt, the risk of infection is linked to raising livestock near irrigation canals and drinking water from small water channels where *Lymnaeid* vectors are colonizing [19]. Egypt has a high infection rate (43.5%) of the *Fasciola* intermediate host snail species [20], increasing the likelihood of contamination of wild vegetables, cultivated plants, and drinking water [21].

Egypt has arid weather, so it is greatly influenced by climate change. A study on the temperature trends between 1950 and 2017 in eight sites across Egypt revealed that the daily maximum temperature levels increased by 1.3 ± 0.1°C and the daily minimum temperatures rose by 1.3 ± 0.3°C [22]. There is a lack of information regarding climate change’s impact on Egypt’s frequency and distribution of human fascioliasis. This study aimed to evaluate the relationship between climatic conditions and the occurrence of human fascioliasis in Assiut Governorate, which reported an outbreak of acute fascioliasis in 2018 [23].

## Methodology

### Study area and patients

**Fig. 1:**
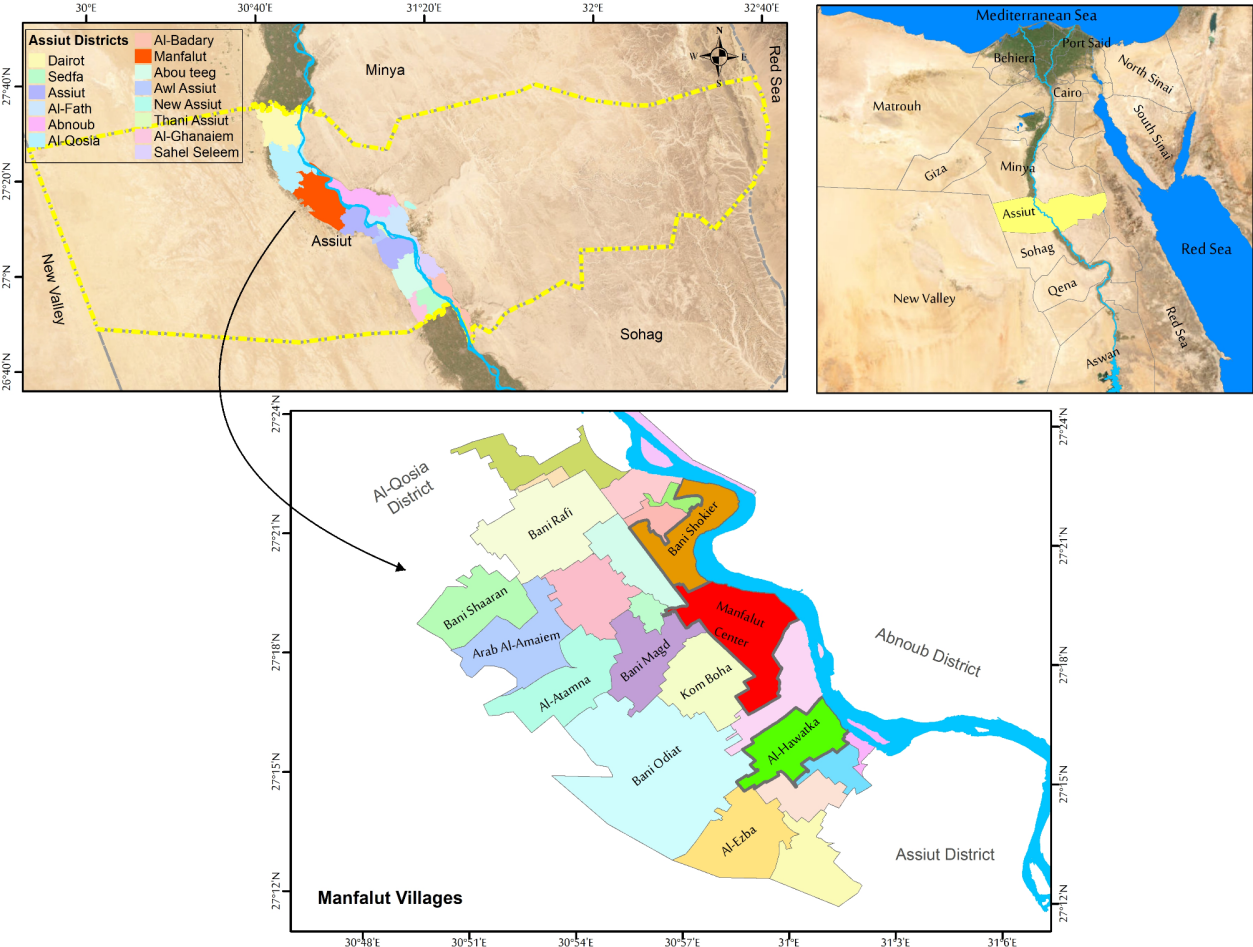
Location map of Assiut Governorate, Egypt, highlights the governorate districts and Manfalut District (where the human fascioliasis outbreak occurred) in red color. Boundaries of districts with the highest cases are bold (Bani Shokier, Manfalut Center, and Al-Hawatka)

The registered cases of *Fasciola* infection in the reports of the Ministry of Health and Population (MOP) from endemic disease clinics in Assiut Governorate and Assiut University Hospital between September 2018 and March 2023 were included in the study. Cases with incomplete data record were excluded. Assiut Governorate is one of the largest governorates in Upper Egypt, located at 27°11′N 31°10′E (Fig. 1). It is located on the west bank of the Nile River, approximately midway between Cairo and Aswan, about 375 km from Cairo. The total population reached 5,126,360 in 2024, according to CAPMAS [24].

*Fasciola* infection was diagnosed based on clinical manifestations, including fever, abdominal pain, jaundice, and hepatomegaly. In addition, hepatic focal lesions or visible worms in common bile ducts on imaging by abdominal ultrasound and/or computed tomography (CT), the presence of peripheral blood eosinophilia in the blood picture, and high titer of serum *Fasciola* antibody with or without *Fasciola* eggs in stool were used to confirm the diagnosis.

Demographic data regarding age, sex, residence, and the distribution among the governorate were recorded. Data regarding the type of clinical presentation of *Fasciola* and response to triclabendazole were also reported which was based on the initial clinical presentation and laboratory or imaging data for each patient.

### Climate datasets

This research used different climate datasets to detect the relation between climatic changes and *Fasciola* frequency in Assiut Governorate. Monthly temperature and humidity data were obtained for the last twenty-three years, from 2000 to 2023. The temperature data was obtained from the fifth-generation European Centre for Medium-Range Weather Forecasts (ECMWF), and atmospheric re-analysis (ERA5). This re-analysis dataset merges model data with observations from across the globe. It is created by applying the fundamental laws of physics, resulting in a complete and coherent dataset that spans several decades. This re-analysis data set provides an accurate description of past climate conditions. Moreover, the specific humidity levels were acquired from the Famine Early Warning Systems Network (FEWS NET) Land Data Assimilation System (FLDAS) dataset from NASA Land Information System at a spatial resolution of 0.01 degree between 2000 and 2023.

### Statistical analysis

Data was analyzed using SPSS version 23. Categorical data were expressed as numbers and percentages, while continuous data were expressed as mean ± SD. Spearman correlation analysis was used to determine the relationship between the number of recorded cases and the air temperature and humidity. A significant p-value was considered if the P-value < 0.05. Furthermore, the Geographic Information Systems (GIS) platform (Arc GIS) was used to analyze the climate data, extract zonal statistics of temperature and humidity data for each district in Assiut Governorate, and study area mapping.

## Results

### Demographic and geographic data of the included patients

The annual prevalence showed that the largest recorded cases were in 2018 when an outbreak was reported in this region. The highest frequency of *Fasciola* was observed in Manfalut district, highlighted on the map. Manfalut district is located on the west bank of the Nile River, in Assiut Governorate, 350 km south of the capital Cairo (Fig. 1).

Reports of three hundred and three *Fasciola* cases were enrolled in the study. Demographic and laboratory data are shown in Table 1. The mean age was 33.9 ± 17.4 years, with the majority having an age range between 21 and 40 years *n* = 132 (43.6%), and females were predominant *n* = 173 (57.1%). Most of the patients were rural residents (*n* = 224, 73.9%). The majority of patients were residents of Manfalut district (89.1%). Acute fascioliasis was recorded in 94.4%, and the majority showed a response to triclabendazole (*n* = 229, 75.6%). *Fasciola* eggs by stool microscopic examination were detected in a few cases *n* = 10 (3.3%).

**Table 1:** Demographic and laboratory data, and the response to treatment of the recorded *Fasciola* cases in the study area from September 2018 to March 2023

Data items	No (%)
**Age in years (mean ± SD)**	33.9 ± 17.4
**(Range)**	(5–75)
**5–20 years**	74 (24.4)
**21–40 years**	132 (43.6)
**41–60 years**	73 (24.1)
**≥** **61 years**	24 (7.9)
**Sex**	
Males	130 (42.9)
Females	173 (57.1)
**Residence**	
Rural	224 (73.9)
Urban	79 (26.1)
**Cases in districts of Assiut Governorate**:	
**Manfalut**	270 (89.1)
**Assiut**	18 (5.9)
**Abnoub**	9 (3)
**Dairut**	3 (1)
**Al-Fath**	2 (0.7)
**Al-Qosia**	1 (0.3)
**Acute fascioliasis**	286 (94.4)
**Response to triclabendazole**	229 (75.6)
**Eosinophilic count: median (IQR)**	3.22 (6.31)
**Eosinophilic percent: median (IQR)**	31 (37.1)
**Detection of diagnostic eggs in stool**	10 (3.3)
**Years of the study period**:	
♣ **From September 2018**	237 (78.2)
♣ **Year 2019**	151 (49.8)
♣ **Year 2020**	121 (39.9)
♣ **Year 2021**	58 (19.1)
♣ **Year 2022**	57 (18.8)
♣ **Up to March 2023**	8 (2.6)

**Data on climate change and its relation to the recorded cases of *Fasciola***:

- **Annual analysis of temperature in relation to cases**.

**Fig. 2:**
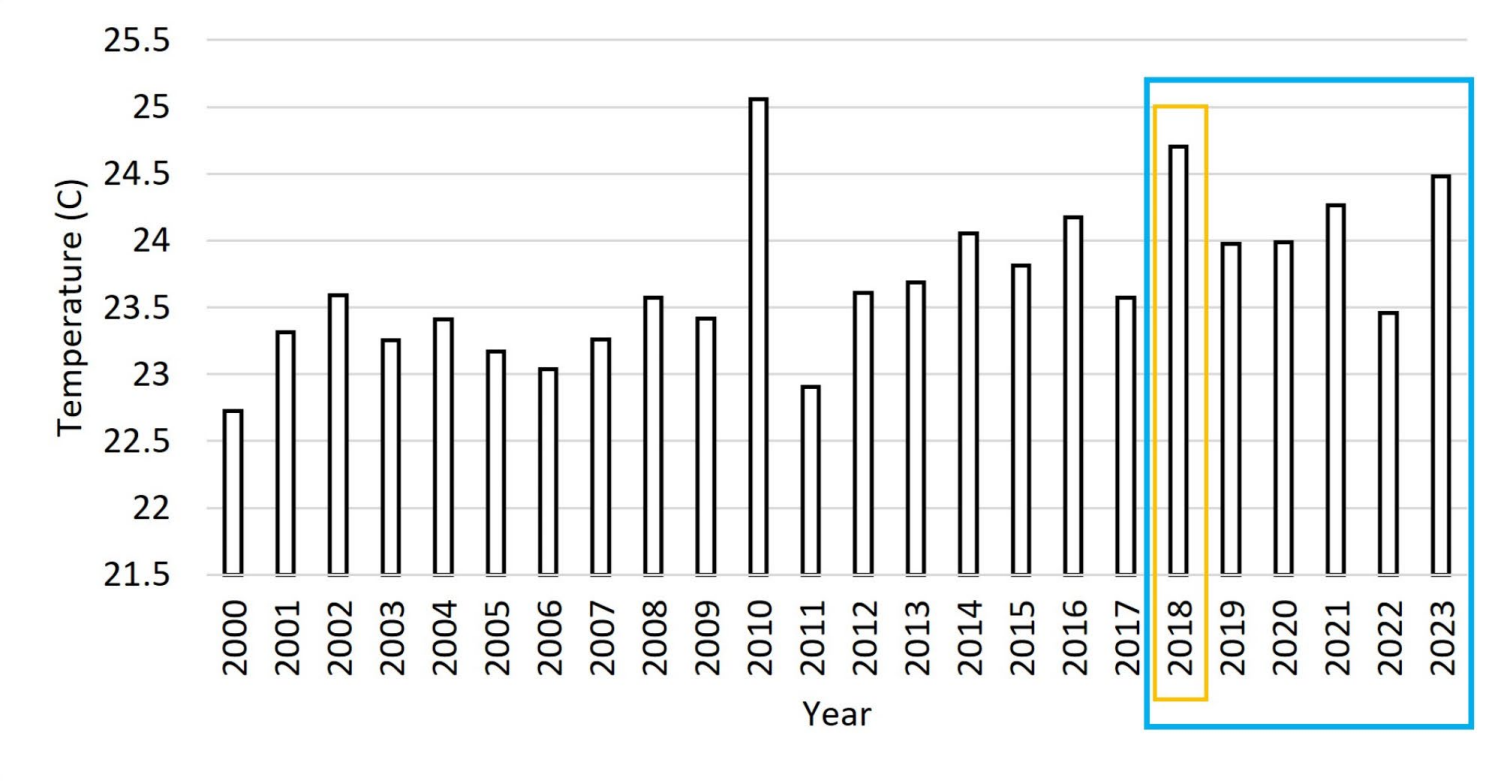
The annual temperature variations in Assiut Governorate from 2000–2023 based on the ERA5 dataset

Figure 2 presents an analysis of the temperature changes between 2000 and 2023. The highest temperatures were recorded in 2010 and 2018. The study focused on the frequency of *Fasciola* infection from 2018 to 2023 and found that the average temperature was the highest in 2018 at around 24.7^°^C, with a maximum of 32.24^°^C in July 2018 and a minimum of 13.48^°^C in January 2018.

The analysis was applied on a lower scale in Assiut districts. It was observed that Manfalut recorded the highest number of *Fasciola* cases during the study period (S1 Fig).

- **Monthly analysis of temperature in relation to cases**.

**Fig. 3:**
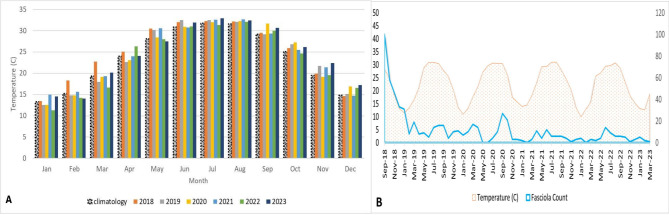
The monthly temperature distribution in Assiut Governorate, A: from 2018 to 2023, and the climatology represents the average temperature from 2000–2017, B: from September 2018 to March 2023

Figure 3A illustrates the monthly temperature changes between 2000 and 2017 and their relation to the temperature in the years of interest in the study when cases were recorded from 2018 to 2023. It can be observed that the temperature exceeded the average climatology values in most of the months, particularly in 2018.

Figure 3B displays the monthly temperature and distribution of *Fasciola* recorded cases in Assiut Governorate from September 2018 to March 2023. The analysis of Fig. 3B suggests that the highest increase in cases occurred during peak temperature in September 2018, decreased with the decline in temperature up to February 2019, and then re-increased up to March 2019 with the re-increase in the temperature. After that, a fluctuation in the recorded cases was seen up to March 2020, with a marked rise in recorded cases coinciding with the rise in the temperature in January 2020. Similarly, a rise in recorded cases was observed during rising temperatures between March and April and between September and October 2020, with the highest peak of recorded cases in September 2020. April to October 2021 and June to October 2022 observations indicated an association between high temperatures and increased *Fasciola* recorded cases. Also, the peak in cases in June 2021 and 2022 corresponded to the highest temperature recorded in June.

- **Annual analysis of humidity in relation to cases**.

**Fig. 4:**
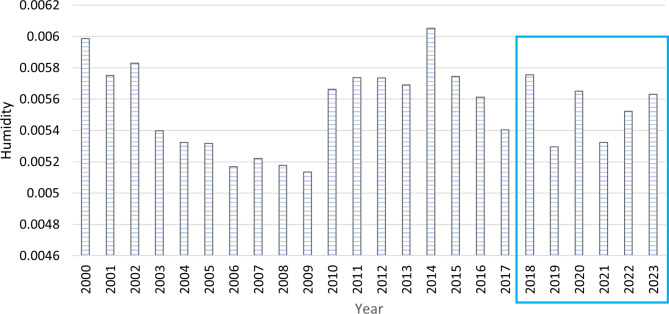
The annual variation in humidity in Assiut Governorate for the period 2000–2023

The influence of humidity changes on *Fasciola* cases was investigated in Assiut Governorate by examining annual and monthly humidity variations. Figure 4 shows the annual variation in humidity from 2000 to 2023. The year 2018 showed the highest humidity levels of any year between 2018 and 2023.

A local scale study investigated the relation between atmospheric humidity and *Fasciola* recorded counts in Assiut districts. The study found a strong positive correlation between humidity and *Fasciola* counts, particularly in the four districts with the highest recorded cases: Manfalut Center, Al-Hawatka, Bani Shokier, and Gimris (*r* = 0.77, *p* = 0.04). These four districts are all part of the Manfalut district (Fig. S2 A, B).

- **Monthly analysis of humidity in relation to cases**.

**Fig. 5:**
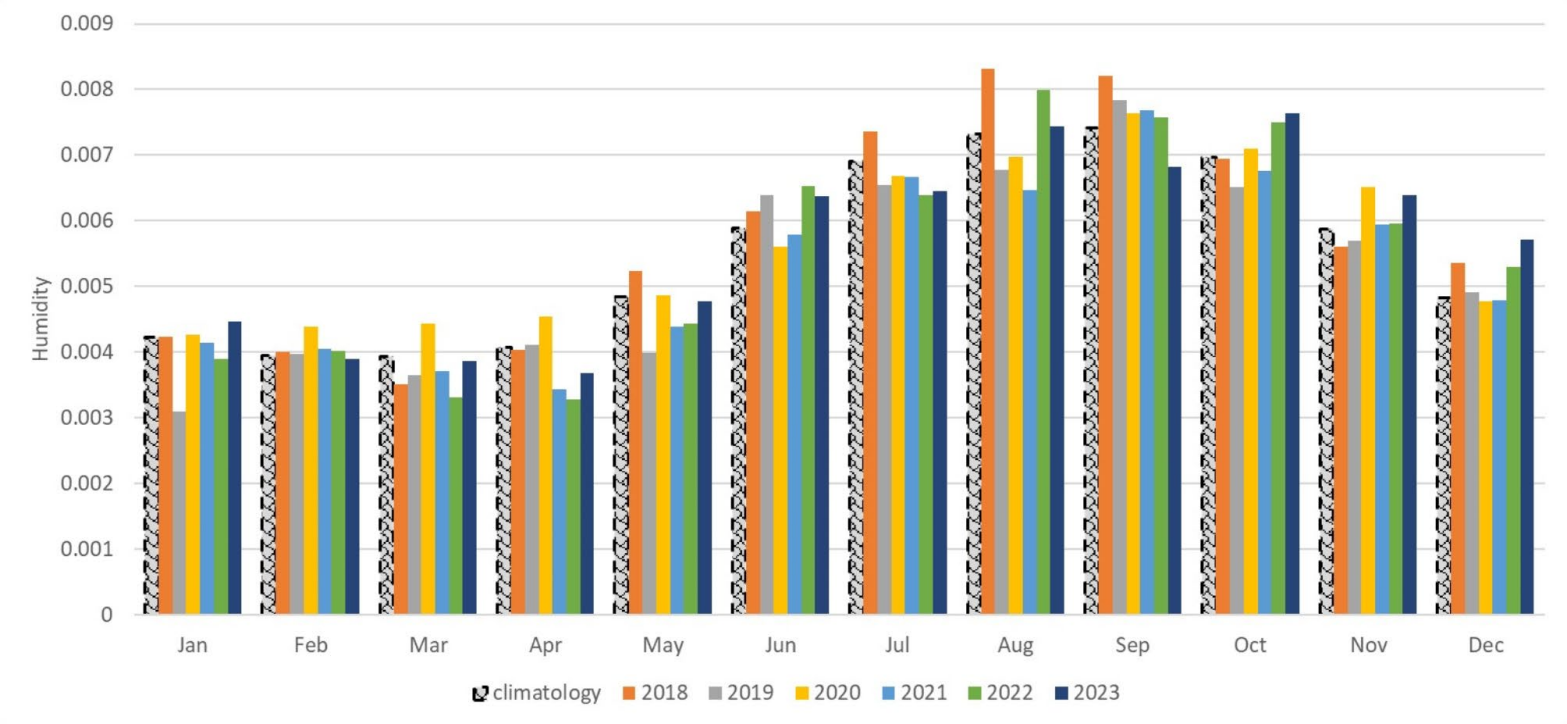
Monthly distribution of humidity in Assiut Governorate for 2018–2023, and climatology data represents the average humidity for 2000–2017

**Fig. 6:**
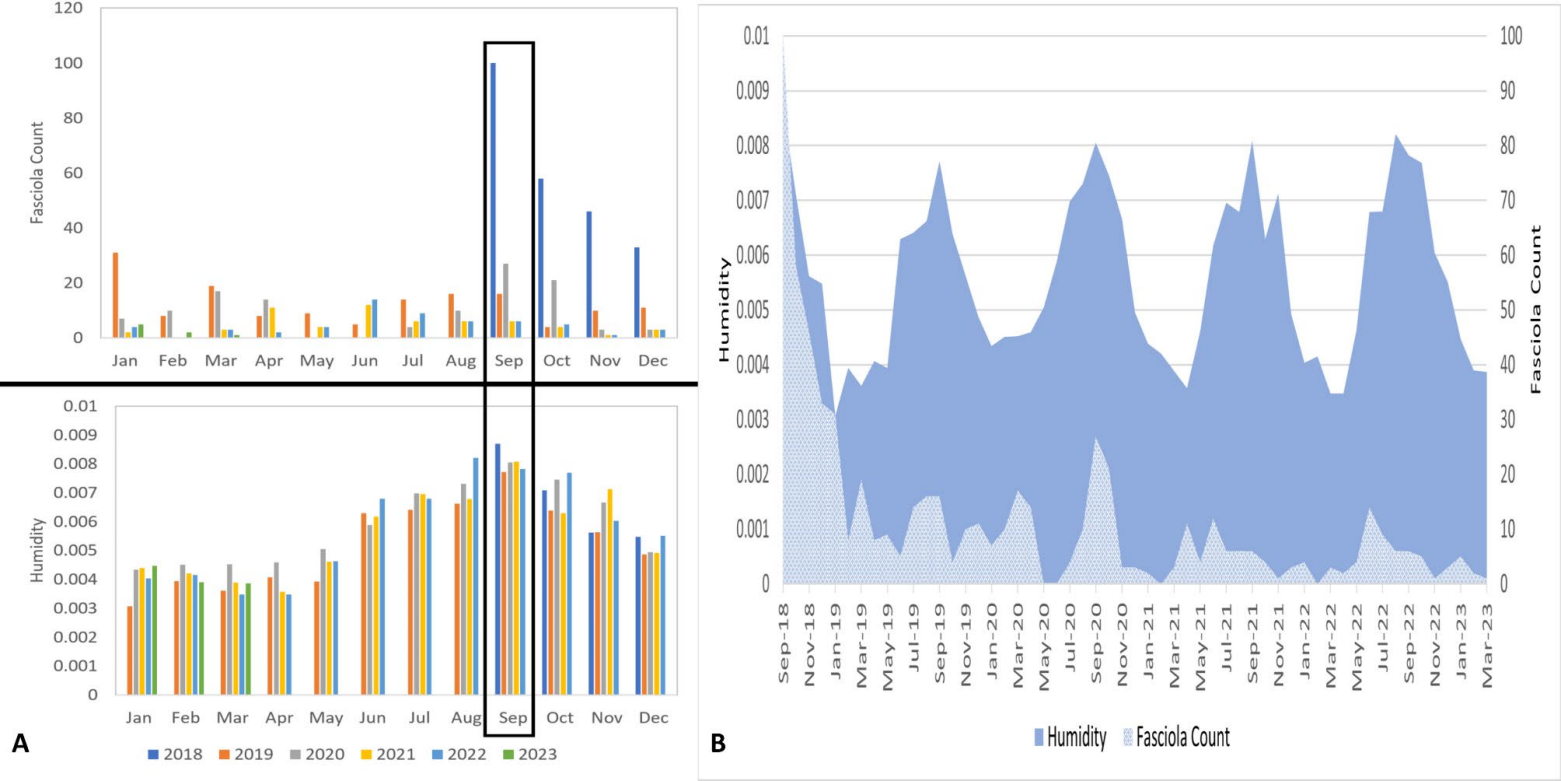
Records of *Fasciola* cases and humidity levels, A: between 2018–2023, B: Monthly distribution of humidity and *Fasciola* recorded counts between September 2018 and March 2023

The humidity levels during 2018–2023 were higher than the average recorded from 2000 to 2017, particularly from April to October 2018 (Fig. 5). During the investigation, the highest humidity level detected was 0.0087 in September 2018, representing the month with the highest recorded cases of Fasciola during 2018–2023. By analyzing the subsequent monthly data, it was found that other small, not significant notches of the rise of the recorded cases occurred between July to October 2020 and May to October 2022, coinciding with high humidity levels (Fig. 6A, B).

Table 2 shows a strong positive correlation between temperature and the recorded cases of *Fasciola* in 2018, 2020, 2021, and 2022 (*r* = 0.92, 0.41, 0.61, and 0.60, respectively). On the other hand, a non-significant negative correlation was detected in the year 2019 and the year 2023. In 2023, the correlation was negative as the analysis was applied to the winter months from January to March due to *Fasciola* data availability; during these months, the study area recorded lower temperatures.

**Table 2:** Relationships between the annually recorded *Fasciola* cases and the atmospheric temperature and humidity during the study period between September 2018 to March 2023

Year	Air temperature		Specific humidity	
	*r* value	*P*-value	*r* value	*P*-value
2018	0.92	0.01*	0.97	0.03*
2019	-0.38	0.22	-0.31	0.32
2020	0.41	0.05	0.36	0.24
2021	0.61	0.04*	0.11	0.74
2022	0.60	0.04*	0.49	0.04*
2023	-0.63	0.57	0.98	0.12

* Significant p value

Moreover, in 2018, humidity and Fasciola frequency had a significant strong positive correlation (*r* = 0.97, *p* = 0.03). In contrast, a non-significant negative correlation was found in 2019. A moderate positive correlation was found again in 2020, 2022, and 2023 (*r* = 0.36, 0.49, and 0.98, respectively), with a significant difference in 2022. However, no correlation was found between humidity and *Fasciola* frequency in 2021 (*r* = 0.11).

## **Discussion**

Fascioliasis is a zoonotic disease transmitted by fresh-water molluscs. Climate parameters significantly affect its prevalence in snails, humans, and livestock, making it important to study these effects for better control and prevention [25]. In this study, the patients were mainly rural residents of Assiut Governorate in Upper Egypt. The annual prevalence showed that the largest recorded cases were in 2018, when an outbreak was reported in this region, mainly from Manfalut district. Preventive measures, control programs, and community awareness led to a decline in case numbers up to March 2023. Fascioliasis is linked with rural communities near irrigation canals, where human-made irrigation areas and livestock grazing are common [26]. Assiut Governorate lies near irrigation canals, showing the highest recorded cases from Manfalut Center, Al-Hawatka, Bani Shokier, and Gimris.

The severity of fascioliasis infection is linked to the proximity of people’s homes to water sources inhabited by *Lymnaeid* snails [27]. The abundance of these snails is higher in areas where different species coexist, increasing the risk of disease transmission, as in the Nile Delta of Egypt, where the climate conditions promote the coexistence of *Galba* and *Radix* species [28]. Controlling the snail population is crucial to reducing the incidence of the disease.

The epidemiology of fascioliasis in Upper Egypt has changed significantly in recent years, with a rise in human outbreaks or seasonal epidemics in certain districts as observed in this study [29, 30]. This may be due to global warming, as observed in previous reports in different parts of the world, such as Turkey [31], Ethiopia [32] Peru, and South America [33, 34].

In this study, a higher prevalence of fascioliasis was recorded in females compared to males, which coincides with previous studies conducted in Egypt [19, 21]. However, another systematic review reported lower prevalence rates in females [16], while a former study conducted in Peru showed no significant differences in patients’ gender [35].

In the current study, the prevalence of fascioliasis was significantly influenced by temperature, showing a strong positive correlation with reported cases of *Fasciola* from 2018, 2020, and 2022. The peak temperature coincided with the highest increase in cases in 2018, and this observation was repeated in subsequent years, with temperatures exceeding the average climatology range in the same regions from 2000 to 2017.

These findings are consistent with previous studies in Italy, Ireland, and Mexico, where temperature significantly impacted the prevalence of animal fascioliasis compared to previous years [11, 36, 37]. Mas-Coma et al. found that temperature significantly affects the production and development of cercariae in intermediate snail hosts [38]. Nevertheless, these results disagree with studies in Malaysia and Pakistan, which found no significant correlation between animal fascioliasis prevalence and temperature changes [39, 40]. The conflicting results may be due to significant temperature variations in those areas, with hot summers causing insufficient soil moisture and endangering *Fasciola* intermediate larval stages [4, 41]. These studies were referenced due to a lack of research papers on human fascioliasis.

In the same context, the incidence of *Fasciola* cases is positively correlated with humidity levels. The highest number of cases occurred during the period with the highest recorded humidity (September 2018). Similar findings have been reported in northwest Spain, where humidity and precipitation levels were found to influence the prevalence of *Fasciola* and gastrointestinal nematode infections in sheep [42]. Also, a systematic review of the epidemiology of fascioliasis in Bangladesh showed a significant increase in *Fasciola* infection in rainy seasons with high humidity that favours the growth of intermediate host snails [43].

It was found that the development of *Fasciola* larval stages in snails and snail reproduction are influenced by certain factors such as rainfall, humidity, and temperature. The optimal temperature range for snail development is 22–25ºC, with 55–70% humidity [44]. Infection rates in *Lymnaea* snails are highest during rainy and summer seasons due to increased temperature, humidity, and rainfall [14, 44]. The risk of egg development is highest at around 30 °C, with a development period of 8–10 days. Several studies support the influence of climatic factors on the development of the parasite [42, 45, 46].

Notably, fasciolosis outbreaks show a seasonal pattern with two peak periods of infection in summer and winter [34, 47]. Snails get infected with *Fasciola* in late spring and early summer, and disease levels peak in late autumn or winter [48]. In the present study, most *Fasciola* cases exhibit a seasonal pattern, with infection peaks occurring in hot, humid months, mainly during the summer and autumn. This could be explained by the delayed egg development under unfavourable winter conditions. Once suitable environmental conditions arise, the infection manifests in the host between July and October [49]. Similar findings were observed in northeastern Punjab, Pakistan, where infection rates of human fascioliasis were significantly higher in the summer and autumn than in the winter and spring [50].

Seasonality of human infections of fascioliasis was found to be frequently associated with heavy rainfall years, as in Western Europe, where the disease occurs mainly during autumn (80.9% of cases) [51]. Nevertheless, sporadic infections could occur throughout the year due to the infective metacercariae’s long survival [52]. Additionally, it was noted that human cases tend to occur during the watercress season from October to April [34].

In Egypt, human fascioliasis is described as an emerging zoonotic disease with a seasonal variation, with an infection peak occurring in August [34]. The summer/autumn outbreaks observed in the present study could be linked to the increased participation of rural residents in field activities such as irrigation. Many farmers and their families wash their animals in canals along with vegetables, clothing, and utensils, particularly in warmer seasons [26]. Consequently, people often consume contaminated vegetables and water from these utensils. This aligns with the increase in temperature and humidity, leading to a monthly peak in *Fasciola* cases that occurred in September 2018, between July-October in 2020, and between May and October 2022.

Egypt’s subtropical location results in varied weather conditions. Lower Egypt has mild winters with some rain, while Upper Egypt has a dry climate with warm days and cold nights. Summers are consistently hot and dry throughout the country [53]. Therefore, rainfall data was not considered significant during the peak months of *Fasciola* due to the hot and dry climate. So, the increase in *Fasciola* cases was primarily attributed to temperature and humidity fluctuations.

The study provides the first evidence of a correlation between climate change and human fascioliasis outbreaks in Upper Egypt. However, it has limitations, such as the retrospective nature of the study causing incomplete data of the patients, lack of snail population assessment, and lack of other environmental parameters that could affect the life cycle of *Fasciola*.

Consequently, it is encouraged to create a climate-based risk map of *Fasciola* epidemics using various forecast risk models to predict future outbreaks in humans and animals and properly manage control approaches. While the study provides correlative evidence of a potential causal relationship between climate change and infection rates, it emphasizes the need for further research to confirm a causal link. In addition, it is recommended to use more sensitive and specific diagnostic techniques, such as molecular methods, to improve the accuracy of case identification in future studies.

## Conclusions

The transmission of human fascioliasis is rising in Upper Egypt and is influenced by local climate characteristics. The study found a strong positive correlation between temperature and humidity with the frequency of the recorded *Fasciola* cases, particularly in 2018 and the following years until 2023. Cases of *Fasciola* exhibit a seasonal pattern, with infection peaks occurring in hot and humid months, mainly in the summer and autumn seasons. Warmer temperatures and high humidity promote transmission and survival rates of parasitic disease, while high humidity levels create favorable conditions for the survival and transmission of *Fasciola* larval stages.

## Electronic supplementary material

Below is the link to the electronic supplementary material.

Supplementary Material 1

Supplementary Material 2

## Data Availability

The data that support the findings of this study are not publicly available. The data are, however, available from the corresponding author upon reasonable request.

## References

[1] Mas-Coma S, Valero MA, Bargues MD. Fasciola, lymnaeids and human fascioliasis, with a global overview on disease transmission, epidemiology, evolutionary genetics, molecular epidemiology and control. Adv Parasitol. 2009;69:41–146. 19622408 10.1016/S0065-308X(09)69002-3

[2] Afshan K, Fortes-Lima CA, Artigas P, Valero MA, Qayyum M, Mas-Coma S. Impact of climate change and man-made irrigation systems on the transmission risk, long-term trend and seasonality of human and animal fascioliasis in Pakistan. Geospat Health. 2014;8(2):317–34. 24893010 10.4081/gh.2014.22

[3] CDC, Fascioliasis. https://www.cdc.gov/dpdx/fascioliasis/index.html

[4] Rapsch C, Dahinden T, Heinzmann D, Torgerson PR, Braun U, Deplazes P, Hurni L, Bär H, Knubben-Schweizer G. An interactive map to assess the potential spread of Lymnaea truncatula and the free-living stages of Fasciola hepatica in Switzerland. Vet Parasitol. 2008;154(3–4):242–9. 18495343 10.1016/j.vetpar.2008.03.030

[5] Malone J. Biology-based mapping of vector-borne parasites by geographic information systems and remote sensing. Parassitologia. 2005;47(1):27. 16044674

[6] Fuentes MV. Proposal of a geographic information system for modeling zoonotic fasciolosis transmission in the Andes. Parasitología Latinoam. 2004;59(1–2):51–5.

[7] Torgerson P, Claxton J. Epidemiology and control. Fasciolosis. 1999;113:149.

[8] Yatoo M, Kumar P, Dimri U, Sharma M. Effects of climate change on animal health and diseases. Int J Livest Res. 2012;2(3):15–24.

[9] Caminade C, McIntyre KM, Jones AE. Impact of recent and future climate change on vector-borne diseases. Ann N Y Acad Sci. 2019;1436(1):157–73. 30120891 10.1111/nyas.13950 PMC6378404

[10] Modabbernia G, Meshgi B, Kinsley AC. Climatic variations and Fasciola: a review of impacts across the parasite life cycle. Parasitol Res. 2024;123(8):1–14. 10.1007/s00436-024-08319-6 39145846

[11] Bosco A, Rinaldi L, Musella V, Amadesi A, Cringoli G. Outbreak of acute fasciolosis in sheep farms in a Mediterranean area arising as a possible consequence of climate change. Geospat Health. 2015;9(2):319–24. 25826313 10.4081/gh.2015.354

[12] De Waal T, Relf V, Good B, Gray J, Murphy T, Forbes A, Mulcahy G. *Developing models for the prediction of Fasciolosis in Ireland*. in *Making science work on the farm: a workshop on decision support systems for irish agriculture*. 2007.

[13] Van Dijk J, Sargison N, Kenyon F, Skuce P. Climate change and infectious disease: helminthological challenges to farmed ruminants in temperate regions. Animal. 2010;4(3):377–92. 22443942 10.1017/S1751731109990991

[14] Dube A, Kalinda C, Manyangadze T, Mindu T, Chimbari MJ. Effects of temperature on the life history traits of intermediate host snails of fascioliasis: a systematic review. PLoS Negl Trop Dis. 2023;17(12):e0011812. 38048345 10.1371/journal.pntd.0011812 PMC10721167

[15] Curtalei F, El Wakeel HAMMOUDZE, Mas-Coma A S, Savioli L. Human Fascioliasis, an emerging public health problem in the Nile Delat, Egypt. 2000; 29: 129–134.

[16] Rosas-Hostos Infantes LR, Paredes Yataco GA, Ortiz-Martínez Y, Mayer T, Terashima A, Franco-Paredes C, Gonzalez-Diaz E, Rodriguez-Morales AJ, Bonilla-Aldana DK. Vargas Barahona L. The global prevalence of human fascioliasis: a systematic review and meta-analysis. Therapeutic Adv Infect Disease. 2023;10:20499361231185413. 10.1177/20499361231185413 PMC10331341 37434654

[17] Mera y Sierra R, Agramunt VH, Cuervo P, Mas-Coma S. Human fascioliasis in Argentina: retrospective overview, critical analysis and baseline for future research. Parasites Vectors. 2011;4:1–18. 21663691 10.1186/1756-3305-4-104 PMC3141741

[18] Caravedo MA, Cabada MM. Human fascioliasis: current epidemiological status and strategies for diagnosis, treatment, and control. Research and Reports in Tropical Medicine. 2020: 149–158. 10.2147/RRTM.S237461 PMC7705270 33273878

[19] Periago MV, Valero MA, Artigas P, Agramunt VH, Bargues MD, Curtale F, Mas-Coma S. Very high fascioliasis intensities in schoolchildren from Nile Delta Governorates, Egypt: The Old World highest burdens found in lowlands. Pathogens. 2021;10(9):1210. 34578242 10.3390/pathogens10091210 PMC8470878

[20] Hadebe MI, Manyangadze T, Kalinda C, Mindu T, Chimbari MJ. Infection rates of Fasciola Intermediate Host Snail Species and their distribution in Africa: a systematic review and Meta-analysis. Trop Med Infect Disease. 2023;8(10):467. 37888595 10.3390/tropicalmed8100467 PMC10610779

[21] Esteban J-G, Gonzalez C, Curtale F, Munoz-Antoli C, Valero MA, Bargues MD, El Sayed M, El Wakeel AA, Abdel-Wahab Y, Montresor A. Hyperendemic fascioliasis associated with schistosomiasis in villages in the Nile Delta of Egypt. Am J Trop Med Hyg. 2003;69(4):429–37. 14640504

[22] Mostafa AN, Wheida A, El Nazer M, Adel M, El Leithy L, Siour G, Coman A, Borbon A, Magdy AW, Omar M. Past (1950–2017) and future (– 2100) temperature and precipitation trends in Egypt. Weather Clim Extremes. 2019;26:100225.

[23] Ahmad AA, Ramadan HK-A, Hassan WA, Hakami MA, Huseein EAM, Mohamed SA-A, Mohamed AA, Elossily NA. New perspectives for fascioliasis in Upper Egypt’s new endemic region: sociodemographic characteristics and phylogenetic analysis of Fasciola in humans, animals, and lymnaeid vectors. PLoS Negl Trop Dis. 2022;16(12):e0011000. 36576925 10.1371/journal.pntd.0011000 PMC9797099

[24] CAPMAS. Central Agency for Public Mobilization and Statistics – Population counts. 2024; apmas.gov.eg.

[25] Freitas DF, Martins IVF, dos Santos AR. Climate change on the forecasted risk of bovine fasciolosis in Espírito Santo state, Brazil. Semina: Ciências Agrárias. 2014;35(6):3147–60.

[26] Mas-Coma S, Bargues M, Valero M. Human fascioliasis infection sources, their diversity, incidence factors, analytical methods and prevention measures. Parasitology. 2018;145(13):1665–99. 29991363 10.1017/S0031182018000914

[27] Mas-Coma M, Esteban J, Bargues M. The traditional epidemiological picture of human fascioliasis has changed markedly in recent years, as outlined below. World Heal Organ. 1999;77:340–6. PMC2557647 10327713

[28] Nukeri S, Malatji MP, Sengupta ME, Vennervald BJ, Stensgaard A-S, Chaisi M, Mukaratirwa S. Potential hybridization of Fasciola hepatica and F. Gigantica in Africa—A Scoping Review. Pathogens. 2022;11(11):1303. 36365054 10.3390/pathogens11111303 PMC9695073

[29] Mekky MA, Tolba M, Abdel-Malek MO, Abbas WA, Zidan M. Human fascioliasis: a re-emerging disease in Upper Egypt. Am J Trop Med Hyg. 2015;93(1):76. 25870421 10.4269/ajtmh.15-0030 PMC4497909

[30] Ramadan HK-A, Hassan WA, Elossily NA, Ahmad AA, Mohamed AA, Abd-Elkader AS, Abdelsalam EMN, Khojah HM. Evaluation of nitazoxanide treatment following triclabendazole failure in an outbreak of human fascioliasis in Upper Egypt. PLoS Negl Trop Dis. 2019;13(9):e0007779. 31553716 10.1371/journal.pntd.0007779 PMC6779272

[31] Beyhan YE, Yılmaz H. Seroprevalence of fascioliasis in the eastern region of Turkey: an eight-year investigation. Turkish J Gastroenterol. 2020;31(11):746. 10.5152/tjg.2020.19243 PMC7759228 33361036

[32] Bekana T, Berhe N, Eguale T, Aemero M, Medhin G, Tulu B, G/hiwot Y, Liang S, Hu W, Erko B. Prevalence and factors associated with intestinal schistosomiasis and human fascioliasis among school children in Amhara Regional State, Ethiopia. Trop Med Health. 2021;49:1–11. 33971981 10.1186/s41182-021-00326-y PMC8111779

[33] Marcos L, Romani L, Florencio L, Terashima A, Canales M, Nestares J, Huayanay L, Gotuzzo E. Hyperendemic and mesoendemic zones of Fasciola infection surrounding urban Lima: an emerging disease? Revista De Gastroenterologia Del Peru. Volume 27. Organo Oficial de la Sociedad de Gastroenterologia del Peru; 2007. pp. 31–6. 1. 17431434

[34] Mas-Coma S. Human fascioliasis: epidemiological patterns in human endemic areas of South America, Africa and Asia. Southeast Asian J Trop Med Public Health. 2004;35(Suppl 1):1–11.

[35] González LC, Esteban JG, Bargues MD, Valero MA, Ortiz P, Náquira C, Mas-Coma S. Hyperendemic human fascioliasis in Andean valleys: an altitudinal transect analysis in children of Cajamarca province. Peru Acta Trop. 2011;120(1–2):119–29. 21767521 10.1016/j.actatropica.2011.07.002

[36] Selemetas N, de Waal T. Detection of major climatic and environmental predictors of liver fluke exposure risk in Ireland using spatial cluster analysis. Vet Parasitol. 2015;209(3–4):242–53. 25777048 10.1016/j.vetpar.2015.02.029

[37] Villa-Mancera A, Reynoso-Palomar A. High prevalence, potential economic impact, and risk factors of Fasciola hepatica in dairy herds in tropical, dry and temperate climate regions in Mexico. Acta Trop. 2019;193:169–75. 30844375 10.1016/j.actatropica.2019.03.005

[38] Mas-Coma S, Valero MA, Bargues MD. Climate change effects on trematodiases, with emphasis on zoonotic fascioliasis and schistosomiasis. Vet Parasitol. 2009;163(4):264–80. 19375233 10.1016/j.vetpar.2009.03.024

[39] Muhammad Faez A, Ahmad Najib M, Noraini AG, Weng Kin W, Abd Rahman A. Wan nor Amilah WAW, & Noor Izani NJ. Seasonal Occurrence of Cattle Fascioliasis in Kelantan, Malaysia. Veterinary Sci. 2023;10(3):202. 10.3390/vetsci10030202 PMC10058272 36977241

[40] Qureshi A, Tanveer A, Maqbool A, Niaz S. Seasonal and monthly prevalence pattern of fasciolosis in buffaloes and its relation to some climatic factors in northeastern areas of Punjab, Pakistan. 2012.

[41] Suhardono, Roberts J, Copeman D. The effect of temperature and humidity on longevity of metacercariae of Fasciola gigantica. Trop Anim Health Prod. 2006;38:371–7. 17165607 10.1007/s11250-006-4310-y

[42] Martínez-Valladares M, Robles-Pérez D, Martínez-Pérez JM, Cordero-Pérez C, Famularo MR, Fernández-Pato N, González-Lanza C, Castañón-Ordóñez L, Rojo-Vázquez FA. Prevalence of gastrointestinal nematodes and Fasciola hepatica in sheep in the northwest of Spain: relation to climatic conditions and/or man-made environmental modifications. Parasites Vectors. 2013;6:1–9. 24289489 10.1186/1756-3305-6-282 PMC3849522

[43] Mia MM, Hasan M, Chowdhury MR. A systematic review and meta-analysis on prevalence and epidemiological risk factors of zoonotic fascioliasis infection among the ruminants in Bangladesh. Heliyon. 2021; 7(12). 10.1016/j.heliyon.2021.e08479 PMC8645451 34917794

[44] Islam K, Islam M, Rauf S, Khan A, Hossain M, Sarkar S, Rahman M. Effects of climatic factors on prevalence of developmental stages of Fasciola gigantica infection in Lymnaea snails (Lymnaea auricularia var rufescens) in Bangladesh. Arch Razi Inst. 2015;70(3):187–94.

[45] Tanveer A. The effects of temperature on survival, growth and reproduction of Bellamaya bengalensis Lamarck, in the laboratory. Punjab Univ J Zool. 1990;5:57–79.

[46] Imani-Baran A, Yakhchali M, Malekzadeh-Viayeh R, Farahnak A. Seasonal and geographic distribution of cercarial infection in Lymnaea Gedrosiana (Pulmunata: Lymnaeidae) in north west Iran. Iran J Parasitol. 2013;8(3):423. 24454436 PMC3887244

[47] Altizer S, Dobson A, Hosseini P, Hudson P, Pascual M, Rohani P. Seasonality and the dynamics of infectious diseases. Ecol Lett. 2006;9(4):467–84. 16623732 10.1111/j.1461-0248.2005.00879.x

[48] Fox NJ, White PC, McClean CJ, Marion G, Evans A, Hutchings MR. Predicting impacts of climate change on Fasciola hepatica risk. PLoS ONE. 2011;6(1):e16126. 21249228 10.1371/journal.pone.0016126 PMC3018428

[49] Luzón-Peña M, Rojo-Vázquez F, Gómez-Bautista M. The overwintering of eggs, intramolluscal stages and metacercariae of Fasciola hepatica under the temperatures of a Mediterranean area (Madrid, Spain). Vet Parasitol. 1994;55(1–2):143–8. 7886913 10.1016/0304-4017(94)90065-5

[50] Qureshi AW, Tanveer A, Mas-Coma S. Epidemiological analysis of human fascioliasis in northeastern Punjab. Pakistan Acta Trop. 2016;156:157–64. 26763724 10.1016/j.actatropica.2015.12.023

[51] Garcia Rodriguez J, Martin Sanchez A, Fernández Gorostarzu J, García Luis E. Fascioliasis in Spain: a review of the literature and personal observations. Eur J Epidemiol. 1985;1:121–6. 3916096 10.1007/BF00141804

[52] Valero MA, Mas-Coma S. Comparative infectivity of Fasciola hepatica metacercariae from isolates of the main and secondary reservoir animal host species in the Bolivian Altiplano high human endemic region. Folia Parasitol. 2000;47(1):17–22. 10.14411/fp.2000.004 10833011

[53] Elmenoufy H, Morsy M, Eid M, El Ganzoury A, El-Hussainy F, Wahab MA. Towards enhancing rainfall projection using bias correction method: case study Egypt. IJSRSET. 2017;3(6):187–94.

