# Supplementary figures and images for: Significance of climate change in the emergence of human fascioliasis in Upper Egypt
Source: Trop Dis Travel Med Vaccines. 2024 Dec 1;10:24. doi: 10.1186/s40794-024-00234-z (PMC11608467; doi:10.1186/s40794-024-00234-z)

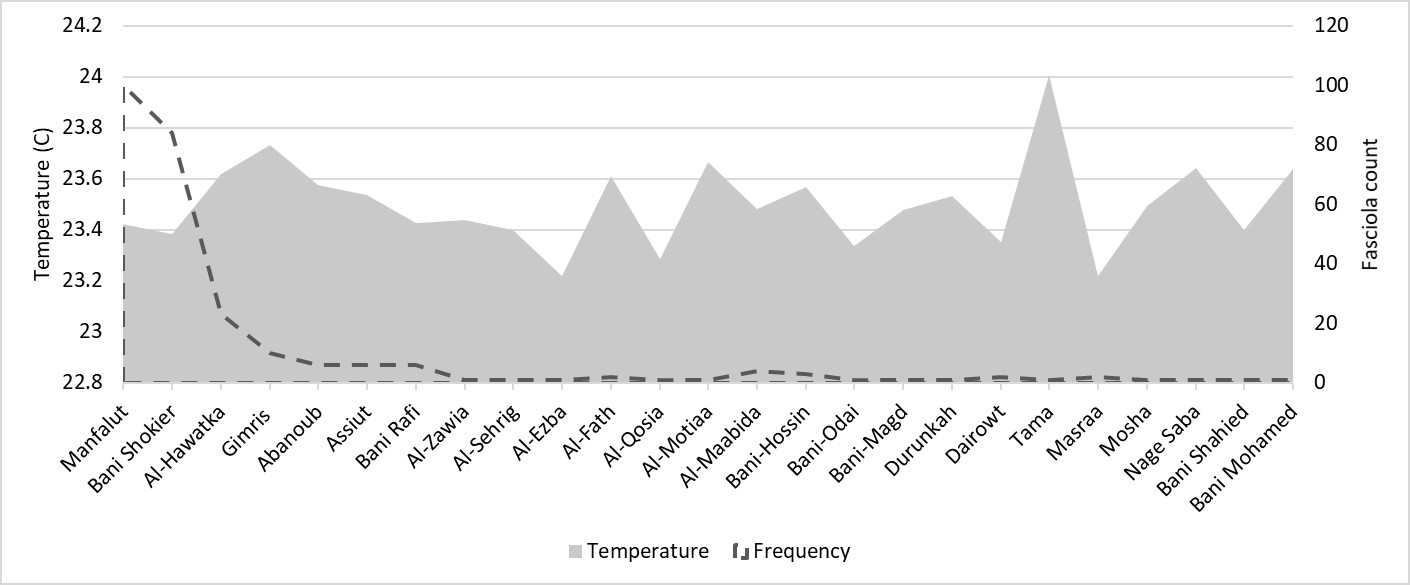

Supplement: Supplementary file 1 — Supplementary Material 1 [file 40794_2024_234_MOESM1_ESM.jpg]

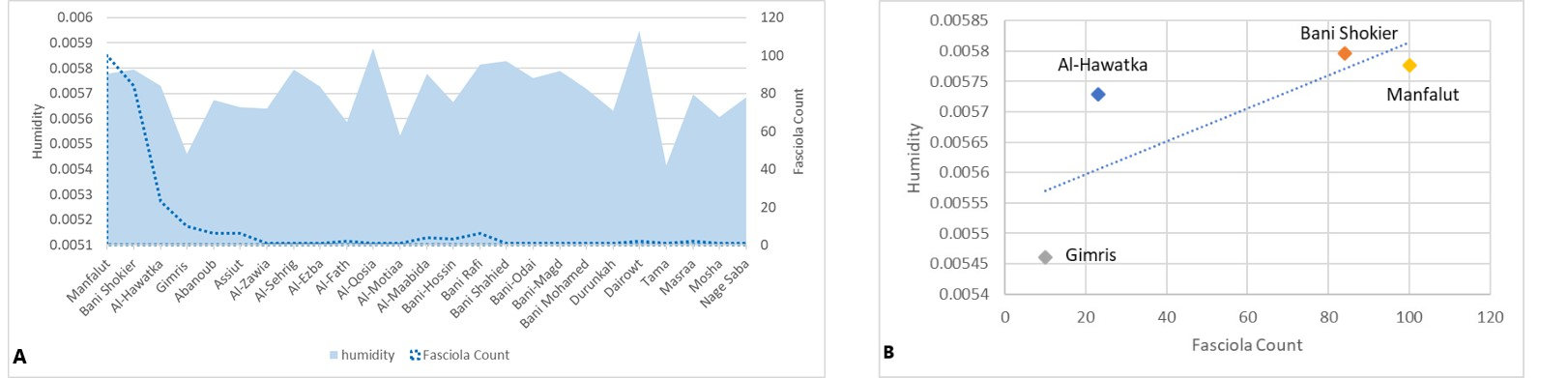

Supplement: Supplementary file 2 — Supplementary Material 2 [file 40794_2024_234_MOESM2_ESM.jpg]
